# Scalp acupuncture for patients with vascular dementia: A protocol for systematic review and meta-analysis of randomized controlled trails

**DOI:** 10.1097/MD.0000000000022798

**Published:** 2020-10-23

**Authors:** Jie Li, Qiuhong Man, Wenchun Wang, Rizhao Pang, Jiancheng Liu, Feng Zhang, Anren Zhang

**Affiliations:** a College of Acupuncture & Massage, Shaanxi University of Chinese Medicine, Shaanxi Key Laboratory of Acupuncture & Medicine, Xixian New Area, Shaanxi Province; b Shanghai Fourth People's Hospital Affiliated to Tongji University School of Medicine; c The General Hospital of Western Theater Command; d Acupuncture and Tuina School, Chengdu University of Traditional Medicine, Chengdu; e Department of Rehabilitation Medicine, Shanghai Fourth People's Hospital Affiliated to Tongji University School of Medicine, Shanghai, China.

**Keywords:** meta-analysis, randomized controlled trails, scalp acupuncture, systematic review, vascular dementia

## Abstract

**Background:**

Vascular dementia (VD) is a kind of acquired intelligence impairment syndrome caused by a series of cerebrovascular factors leading to brain tissue damage. Scalp acupuncture is widely used to treating VD. However, there is no a systematic review has been used to assess the efficacy and safety of scalp acupuncture therapy for VD. Therefore, the purpose of this paper is to systematically evaluate the effects of scalp acupuncture on VD.

**Methods:**

We will search the following databases from their inception to July 2020: PubMed, Chinese National Knowledge Infrastructure (CNKI), Wan Fang Database, Embase, Medline, Chinese Biomedical Literature Database (CBM), EBSCO, Web of Science, Technology Periodical Database (VIP), the Chongqing VIP Chinese Science and Cochrane Library. At the same time, we will retrieve other resources including conference articles, and gray literature. The randomized controlled trials (RCTs) in English or Chinese associated with scalp acupuncture for VD will be included. Our study data collection and analysis will be conducted independently by 2 reviewers, and Rev Man V.5.3.5 statistical software will be used to performing meta-analysis.

**Results:**

This review research will provide a high-quality synthesis to evaluate the efficacy and safety of scalp acupuncture for patients with VD.

**Conclusion:**

This study will provide available evidence to judge whether scalp acupuncture is an effective and safe intervention for patients with VD. It also will provide reliable evidence for its widespread application.

**Ethics and dissemination:**

This systematic review will provide convincing evidence for both patients and clinicians. It does not require ethical approval and the results will be published in a peer-reviewed journal.

**OSF Registration number:**

DOI 10.17605/OSF.IO/7CYZR.

## 1 Introduction

As we all know, Vascular dementia (VD) refers to a kind of intelligence impairment syndrome caused by a series of cerebrovascular factors leading to brain tissue damage.^[1]^ Most of these patients are over 50 years old, and they have the characteristics of stepwise progression and fluctuating course. With the accelerating aging of the population, the prevalence of dementia in China continues to rise, it is estimated that the total number of people with senile dementia and vascular dementia will exceed 16 million by 2030.^[2]^ As an important type of dementia, VD has seriously harmed the physical and mental health of middle-aged and elderly people, which brought economic and spiritual burden to the families and society of patients.^[3,4]^

As a preventable and treatable disease, VD has become the second largest type of dementia in China after Alzheimer's disease.^[5,6]^ Therefore, it is of great significance to find an effective treatment. A large number of studies have shown that VD has an important feature that other types of dementia do not have–reversibility.^[7,8]^ In clinic, modern medicine of VD is lack of specific therapy, but, the traditional Chinese medicine acupuncture and moxibustion has better curative effect, especially early acupuncture intervention has positive clinical significance for VD.^[9–12]^

As an important means of traditional Chinese medicine treatment of VD.^[13,14]^ Scalp acupuncture is a kind of micro acupuncture therapy based on the combination of traditional acupuncture theory and the theory of cortical function localization of scalp projection in Western medicine. Due to the unique position of acupuncture therapy highlights its advantages: safety, simple acupoint selection, simple operation, not easy to induce hysteresis. At present, more and more scalp acupuncture is being used in the treatment of VD.^[15–18]^ However, there is no systematic review at home and abroad to evaluate the efficacy and safety of scalp acupuncture in the treatment of VD. Therefore, this review will assess the efficacy and safety of scalp acupuncture therapy for VD compared with western medicine and other acupuncture therapies. This systematic review will be the first to evaluate the effects of scalp acupuncture on VD, and I hope we can provide convincing results.

## 2 Methods and analysis

### 2.1 Eligible criteria for including studies

#### 2.1.1 Types of studies

The types of studies including all available randomized controlled trials (RCTs) and quasi-randomized controlled trials on scalp acupuncture for people with VD. Others such as case report, retrospective study and studies which refer to importance random study methods will be excluded.

#### 2.1.2 Types of participants

The types of participants including who have been diagnosed with VD, according to at least one of the current or past definitions. People who have been diagnosed with VD include regardless of their age, sex, or race.

#### 2.1.3 Types of intervention and types of comparisons

Our experimental intervention measures should be scalp acupuncture alone. The control group include drugs, body acupuncture therapy, and physical, exercise.

#### 2.1.4 Types of outcome measures

The primary outcome measurements will be improvement in cognitive function and behavioural disturbances those measured by mimi-mental state examination (MMSE), Hasegama^,^s dementia scale (HDS),Hasegawa dementia scale revised (HDS-R), activities of daily living (ADL).

The secondary Outcome Measures include the overall effective rate. Other outcomes included the quality of life (QOL), clinical dementia rating (CDR), Wechsler Memory Scale(WMS), safety and adverse events of scalp acupuncture alone will be observed.

### 2.2 Search methods for identification of studies

We will search the following databases from their inception to July 2020: PubMed, CNKI, WanFang Database, Embase, Medline, CBM, VIP, EBSCO, Web of Science, the Chongqing VIP Chinese Science, and Cochrane Library. In addition, we will manually retrieve other resources including conference articles, and gray literature. The randomized controlled trials (RCTs) in English or Chinese associated with Scalp acupuncture for VD will be included. The research including disease, intervention methods and study types 3 parts: (“dementia” or “Vascular Dementia” or “Vascular” or “cognitive impairment” or “cognitive disorders” or “cognitive deficits” or “vascular cognitive impairment”) and (“The International Standards of the Nomenclature of Scalp Acupuncture Zones” or “Olfactory Three-Needle” or “Xiu san zhen” “Jiao shi” or “Fang shi” or “Zhu shi” or “Jin Three-Needle Therapy”) and (“trial” or “randomly” or “randomized” “controlled clinical trial” or “randomized controlled trial”). The example search strategy PubMed in Table 1 will be used. This search strategy will be used in several other databases.

**Table 1:**
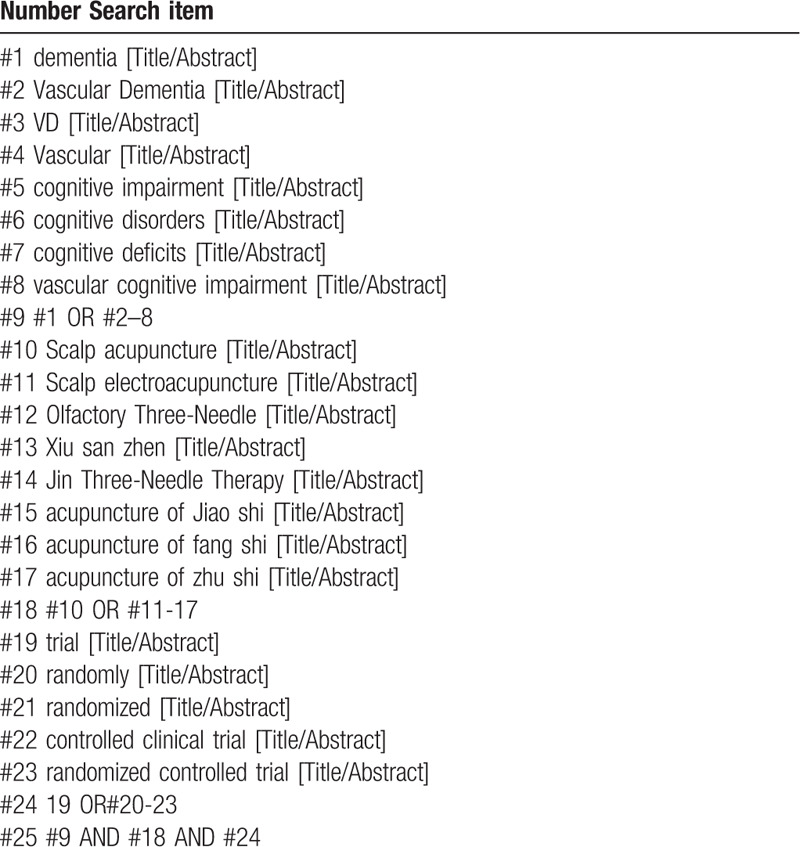
PubMed Search strategy draft.

### 2.3 Data collection and analysis

#### 2.3.1 Data extraction and management

**Figure 1:**
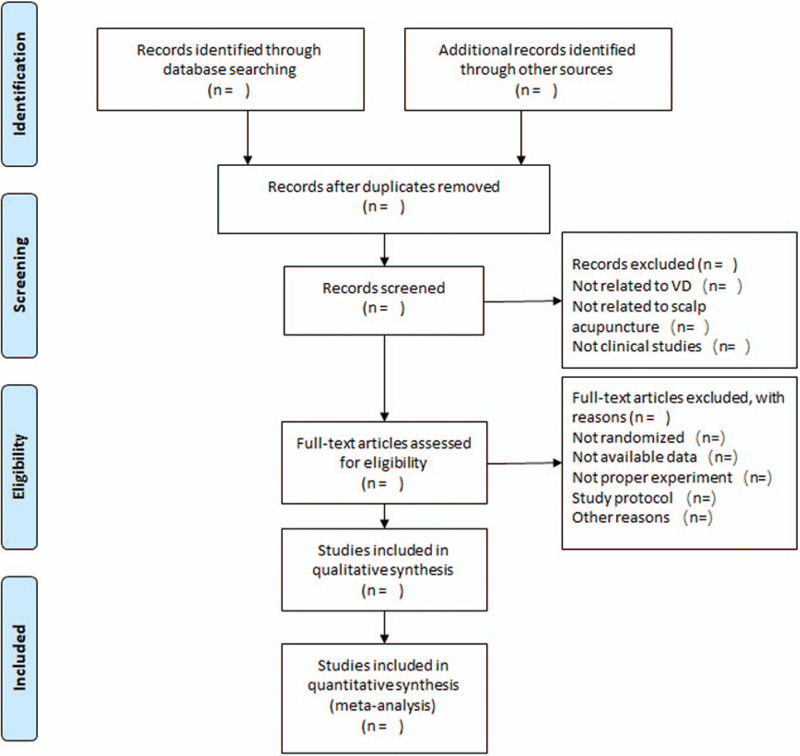
Flow diagram of the study selection process.

After finishing the search work, 2 reviewers (LJ and MQH) will finish the screening process will be independently. After reading titles, abstracts, and full texts. The third reviewer (ZAR) will evaluate whether the studies will be satisfied according to inclusion criteria. This study process is shown in Figure 1 below. The unit of analysis will be conducted by the independent reviewers (PRZ).

#### 2.3.2 Assessment of risk of bias

The bias tool of Cochrane Manual V.5.1.0 is used to conduct the assessment of risk about bias by 2 independent reviewers (LJ and LJC). This process includes: random sequence generation, allocation sequence concealment, blinding, incomplete data, and other sources. Any assessment of the bias has caused controversy will be resolved by the third reviewer (WWC). The biased results will be divided into 3 levels: “low risk of bias” “high risk of bias”, and “uncertain risk of bias”.

#### 2.3.3 Assessment of heterogeneity

The heterogeneity of our research data will be analyzed with *I*^2^ statistic, The trials statistical heterogeneity is significant large when the *I*^2^ value exceeds 50%, and our meta-analysis will not be conducted. In this time, we will carry out sensitive analysis or subgroup stratification analysis to explore the possible reasons of causing heterogeneity.

#### 2.3.4 Dealing with missing data

Any missing data will be complemented by the independent reviewers (PRZ) through contacting with the corresponding author.

#### 2.3.5 Assessment of reporting biases

We will use the funnel to assess reporting biases. We will conduct a test for funnel plot asymmetry using the Egger method if the numbers of available studies are sufficient.

#### 2.3.6 Data synthesis

The Review Manager V.5.3 will be used to analyze the data. The test result indicated little or no heterogeneity. The specific methods are as follows: When the *I*^2^ test is less than 50%, the fixed-effects model will be used for data synthesis. When the *I*^2^ test is between 50% and 75%, the random-effects model will be conducted for data synthesis. When the *I*^2^ test is higher than 75%, the meta-analysis will not be performed. When data cannot be synthesized, we will try to explore the possible reasons, and provide a descriptive analysis to solve this problem.

#### 2.3.7 Subgroup analysis and sensitivity analysis

Subgroup analysis will be used to evaluate high heterogeneity. The factors affecting the heterogeneity include the different combinations of scalp acupuncture, different course time, and other factors. Sensitivity analysis will be used to test the robustness of the main decisions in the review process, which including the impact of quality of methods, sample size, and related issues on the study.

#### 2.3.8 Grading the quality of evidence

We will use the Grading of Recommendations Assessment, Development and Evaluation (GRADE) approach to evaluate the quality of evidence for all results of this systematic review. The quality will be divided into 4 levels: high, moderate, low, or very low.^[19]^

## 3 Discussion

Vascular dementia is equivalent to the category of “stupidity” and “forgetfulness” in traditional Chinese medicine. According to traditional Chinese medicine, the location of the disease is brain, and it is often caused by prolonged illness into collaterals, which can lead to Qi deficiency and blood stasis, block brain orifices, mental retardation, and even dementia, mental disorders and other symptoms. According to the traditional Chinese medicine theory, “The head is the meeting of all the Yang”. Qi and blood are gathered on the head, and the head is closely connected with the meridians and acupoints of the whole body. Scalp acupuncture can directly stimulate the “meeting of all the Yang”, which has the effect of dredging meridians, regulating qi, opening depression, promoting blood circulation, and removing blood stasis.,

After years of research and practice, scalp acupuncture has been explored and summarized. Scalp acupuncture is based on the inheritance of traditional Chinese medicine and acupuncture therapy of world intangible cultural heritage, and it is based on the principle of functional localization of cerebral cortex and acupuncture as a means, which can be used for the treatment of various diseases.^[20]^ It is often used in the treatment of brain-derived diseases. There are many kinds of scalp acupuncture therapy. They include specific head acupoint stimulation, such as Baihui, Taiyang and other acupoints, as well as stimulation of various regions and lines of the head, such as Fang's scalp acupuncture, Jiao's scalp acupuncture, international scalp acupuncture, and so on.^[21]^ In recent years, it has been found that scalp acupuncture has a great effect on increasing the blood supply of cerebral cortex, increasing the metabolic level of brain cells, activating potential neurons, promoting the formation of synapses of brain cells.^[22]^ Moreover, scalp acupuncture is simple, safe and effective. Although the advantages of scalp acupuncture in the treatment of VD are obvious, there is still no systematic review in English at present.

This article will be the first review on the systematic evaluation of scalp acupuncture for VD. It will draw reasonable conclusions by collecting evidence, sorting out and analyzing data about the efficacy and safety of scalp acupuncture in the treatment of VD. We hope this study will provide convincing evidence for both patients and clinicians. In addition, this systematic review also has some limitations. Our research only focuses on articles published in English and Chinese, and no articles in other languages are collected.

## Author contributions

**Data curation:** Qiuhong Man, Feng Zhang.

**Formal analysis:** Jie Li, Wenchun Wang.

**Funding acquisition:** Anren Zhang.

**Investigation:** Wenchun Wang, Anren Zhang.

**Methodology:** Jiancheng Liu.

**Software:** Jiancheng Liu, Feng Zhang.

**Supervision:** Rizhao Pang, Anren Zhang.

**Writing – original draft:** Jie Li.

**Writing – review & editing:** Qiuhong Man.
